# Draft genome sequence data of the multidrug-resistant bacterium *Staphylococcus haemolyticus* 010503B isolated from an aerosol sample in a hospital waiting area in Thailand

**DOI:** 10.1016/j.dib.2024.110154

**Published:** 2024-02-06

**Authors:** Uraiwan Kositanont, Kanjana Changkaew, Manassanan Phatcharaharikarn, Thunwarat Songngamsuk, Ruchirada Changkwanyeun, Montri Yasawong

**Affiliations:** a Faculty of Public Health, Thammasat University, Pathum Thani 12121, Thailand; b Program on Environmental Toxicology, Chulabhorn Graduate Institute, Bangkok 10210, Thailand; c Faculty of Public Health, Thammasat University (Lampang Campus), Lampang 52190, Thailand; d Center of Excellence on Environmental Health and Toxicology (EHT), OPS, MHESI, Bangkok 10400, Thailand

**Keywords:** Multidrug resistance, Aminoglycoside, Beta-lactam, Lincosamide, Macrolide, Streptogramin b, Tetracyclin

## Abstract

*Staphylococcus haemolyticus* 010503B is a multidrug-resistant bacterium isolated from an outpatient clinic in a hospital waiting area in Thailand. Here we present the draft genome sequence of *S. haemolyticus* 010503B. The paired-end reads were generated on the Illumina NextSeq 550 sequencer using genomic DNA from the pure culture of *S. haemolyticus* 010503B. The draft genome consisted of 114 contigs with a total size of 2,457,654 base pairs, an N50 of 57,312 base pairs and a GC content of 32.60%. The dDDH between 010503B and *Staphylococcus haemolyticus* SM 131^T^ was 91.9%, identifying the strain as *Staphylococcus haemolyticus*. The data presented holds promise for bacterial classification, comparative genomics, analysing antimicrobial resistance comprehensively, and assessing bacterial virulence factors of *S. haemolyticus*. The draft genome sequence data has been deposited at NCBI under Bioproject accession number PRJNA550309.

Specifications Table

**Table:** 

Subject	Biological sciences
Specific subject area	Omics: Genomics
Data format	Raw and analysed
Type of data	Tables, figures
Data collection	DNA was extracted using the PureLink^TM^ Genomic DNA Mini Kit and sequenced on an Illumina NextSeq 550. AfterQC v0.9.6 was used for adapter trimming and quality filtering. Genome assembly was performed using Unicycler v0.5.0 and assembly metrics were determined using QUAST v5.0.2. Genome quality was assessed using CheckM v1.1.2. Digital DNA-DNA hybridisations and a phylogenomic tree were analysed using the Type (Strain) Genome Server. Genomic map was generated by Proksee. Genome annotation was performed using the NCBI Prokaryotic Genome Annotation Pipeline. Identification of antimicrobial resistance genes and prediction of resistance phenotypes were performed using ResFinder v4.3.3.
Data source location	*Staphylococcus haemolyticus* 010503B was obtained from an aerosol sample collected in a waiting area at Thammasat University Hospital, located at latitude 14.07341 and longitude 100.61513 in Thailand.
Data accessibility	The sequencing data were deposited in the National Center for Biotechnology Information (NCBI) Genbank database under accession number JAWLJD000000000. The deposited draft genome sequencing data are available at https://www.ncbi.nlm.nih.gov/nuccore/JAWLJD000000000.

## 1 Value of the Data

• The draft genome sequence of *S. haemolyticus* 010503B has potential value for scientific studies in the fields of bacterial taxonomy and ecology, particularly in relation to the identification and distribution of taxa.
• The draft genome sequence of *S. haemolyticus* 010503B provides potential benefits for comparative genomic research on other *Staphylococcus* species with multidrug resistance.
• Elucidating the genome sequence of *S. haemolyticus* 010503B can potentially aid in the identification of antimicrobial resistance genes and the prediction of drug resistance profiles.

## 2 Data Description

**Fig. 1:**
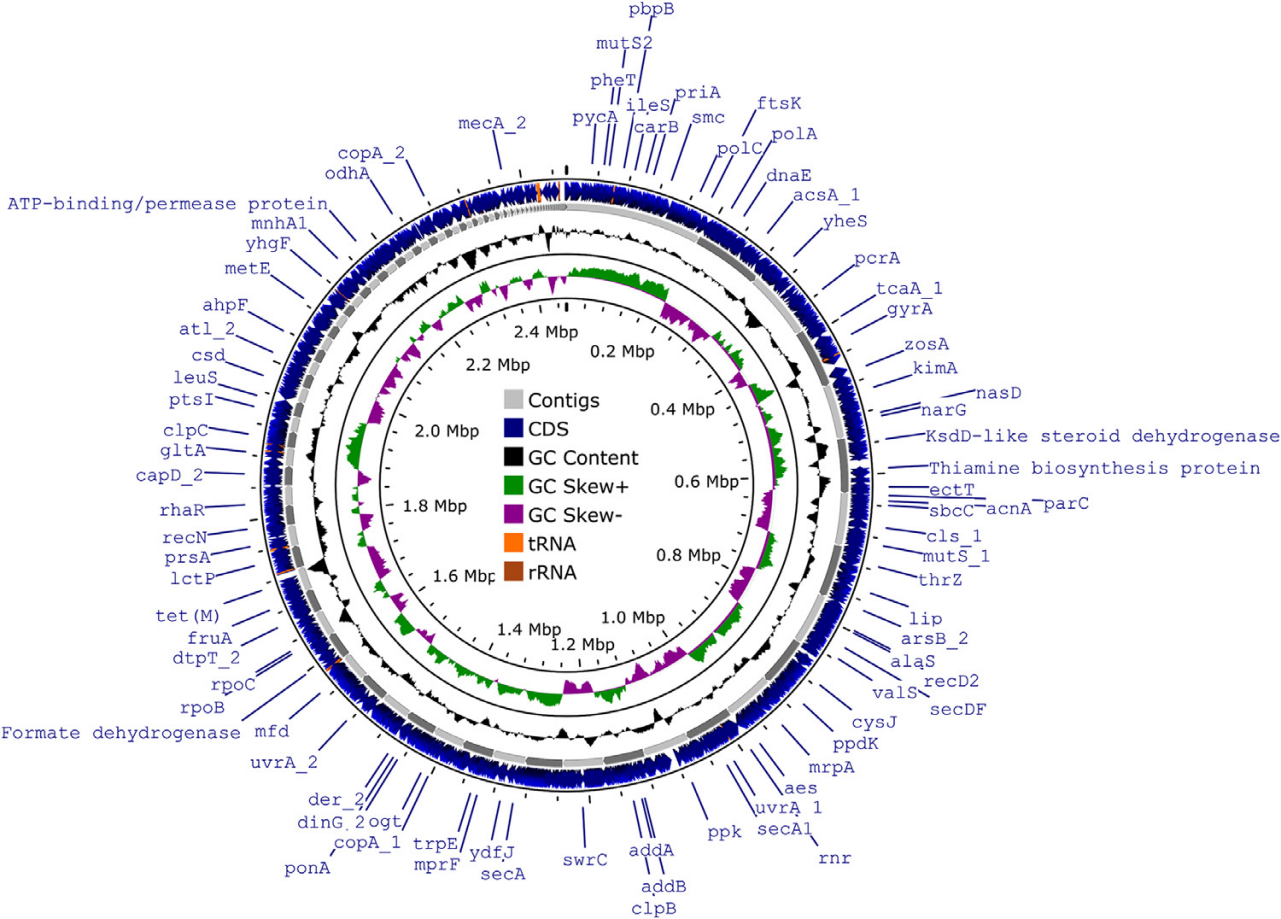
The genome map for *S. haemolyticus* 010503B was generated using Proksee (https://proksee.ca/, accessed 26 October 2023). Blue arrows represent the coding DNA sequences (CDSs), while grey arrows represent the contigs. GC skew+ and GC skew- are represented by green and purple peaks respectively, while black peaks indicate GC content. (For interpretation of the references to color in this figure legend, the reader is referred to the web version of this article.)

Here we present the draft genome sequence data of *S. haemolyticus* 010503B (Fig. 1), including its gene clusters with antimicrobial resistance genes and the predicted of phenotypes.

The genome consisted of 114 contigs with a total size of 2,457,654 bp, an N50 value of 57,312 bp and a GC content of 32.60% (Table 1).

**Table 1:** Genomic features and assembly statistics for *S. haemolyticus* 010503B.

Attribute	*S. haemolyticus* 010503B
Genome size (bp)	2,457,654
Number of contigs	114
Genome coverage	422×
GC content (%)	32.60
Largest contig (bp)	192,842
N50	57,312
N75	27,432
L50	15
L75	31
Total gene	2469
Total CDS	2406
tRNA	52
rRNA	7
ncRNA	4

**Fig. 2:**
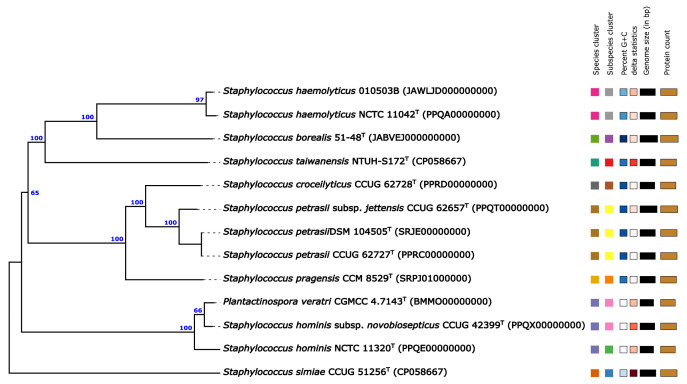
Phylogenomic tree reconstructed using whole-genome sequence data from *S. haemolyticus* 010503B and its closely related type strain on the TYGS platform. Branch numbers were determined based on pseudo-bootstrap support values greater than 60% from 100 replicates using Genome Blast Distance Phylogeny (GBDP), with an average branch support of 88.0%.

The draft genome of *S. haemolyticus* 010503B was found to be 99.45% complete, with an estimated contamination of less than 1%. Furthermore, the digital DNA-DNA hybridisation value between strain 010503B and *Staphylococcus haemolyticus* SM 131^T^ was 91.9%, reliably identifying strain 010503B as a *Staphylococcus haemolyticus* strain. The phylogenomic tree of the strain 010503B and closely related type strains is shown in Fig. 2. Whole genome sequence (WGS)-based antimicrobial susceptibility testing (AST) revealed the existence of recognised multidrug resistance genes with significant similarity, namely *aph(3)-III* (100.00%), *ant(6)-Ia* (99.84%), *aac(6)-aph(2)* (100.00%), *mecA* (99.95%), *blaZ* (95.05–95.90%), *lnu*(A) (99.79%), *erm*(C) (100.00%), *tet*(M) (100.00%), and *tet*(K) (100.00%) within the *S. haemolyticus* 010503B genome (Table 2).

**Table 2:** Identification of antimicrobial resistance genes and prediction of resistance phenotypes from the 010503B genome sequence.

Resistance Gene	Identity (%)	Alignment length/gene length	Position in reference	Contig	Position in contig	Resistance phenotype	Genbank accession number
*aph(3)-III*	100.00	795/795	1–795	76	2,017–2,811	Amikacin, Isepamicin, Kanamycin, Neomycin, Lividomycin, Paromomycin, Ribostamycin, Butirosin	M26832
*ant(6)-Ia*	99.84	642/909	269–909	76	744–1,385	Streptomycin	AF330699
*aac(6)-aph(2)*	100.00	1440/1440	1–1440	85	80–1,519	Gentamicin, Tobramycin, Streptomycin, Amikacin, Isepamicin, Dibekacin, Kanamycin, Netilmicin, Fortimicin	M13771
*mecA*	99.95	2007/2007	1–2007	67	2,600–4,606	Amoxicillin, Amoxicillin+Clavulanic acid, Ampicillin, Ampicillin+Clavulanic acid, Cefepime, Cefixime, Cefotaxime, Cefoxitin, Ceftazidime, Ertapenem, Imipenem, Meropenem, Piperacillin, Piperacillin+Tazobactam	AB505630, BX571856
*blaZ*	95.90	586/801	29–614	106	1–586	Amoxicillin, Ampicillin, Piperacillin, Penicillin	NZ_FHDT01000016
*blaZ*	95.90	586/846	74–659	106	1–586	Amoxicillin, Ampicillin, Piperacillin, Penicillin	FHDH01000010, FGWO01000019, LNTF01000008, NZ_CTTL01000039, NZ_JVAT01000021, NZ_JUUA01000057, IFD01000012, JITV01000006
*blaZ*	95.90	586/801	28–613	107	2–587	Amoxicillin, Ampicillin, Piperacillin, Penicillin	NZ_FHDT01000016
*blaZ*	95.05	586/801	73–658	107	2–587	Amoxicillin, Ampicillin, Piperacillin, Penicillin	FHDH01000010, FGWO01000019, LNTF01000008, NZ_CTTL01000039, NZ_JVAT01000021, NZ_JUUA01000057, JIFD01000012, JITV01000006
*lnu*(A)	99.79	486/486	1–486	77	44–529	Lincomycin	M14039
*erm*(C)	100.00	735/735	1–735	78	332–1,066	Lincomycin, Clindamycin, Erythromycin, Quinupristin, Pristinamycinia, Virginiamycins	M13761
*tet*(M)	100.00	1,920/1,920	1–1,920	27	18,804–20,723	Tetracycline, Doxycycline, Minocycline	AM990992
*tet*(K)	100.00	1,380/1,380	1–1,380	68	1,966–3,345	Tetracycline, Doxycycline	U38656

Prediction of resistance phenotypes suggests that *S. haemolyticus* 010503B may be insusceptible to some antibiotics from six classes of antibiotics, including 1) Aminoglycoside - amikacin, butirosin, dibekacin, fortimicin, gentamicin, isepamicin, kanamycin, lividomycin, netilmicin, neomycin, paromomycin, ribostamycin, streptomycin, and tobramycin, 2) Beta-lactam - amoxicillin, ampicillin, cefepime, cefixime, cefotaxime, cefoxitin, ceftazidime, ertapenem, imipenem, meropenem, and piperacillin, 3) Tetracycline - tetracycline, doxycycline and minocycline, 4) Streptogramin b - pristinamycin ia, quinupristin, and virginiamycin s, 5) Lincosamide - clindamycin and lincomycin, and 6) Macrolide - erythromycin (Table 2). The draft genome sequence could be used for a comprehensive analysis of antimicrobial resistance and bacterial virulence factors of *S. haemolyticus*.

## 3 Experimental Design, Materials and Methods

### 3.1 Bacterial isolation and identification

Aerosol samples were collected using an Andersen six-stage impactor (BGI Inc, Waltham, MA) in the patient waiting area for medical examinations, outpatient clinics at Thammasat University Hospital, Pathum Thani Province, Thailand. An Andersen six-stage impactor with a volume of 28.3 L/min for 15 min (BGI Inc, Waltham, MA) was placed on the floor at a height of 1.5 m. At each stage of the sterilised impactor, a medium plate containing Baird Parker Agar (BPA) (Oxoid, UK) was placed. After aerosol sampling, BPA plates were incubated at 37°C for 48 h. A single colony of strain 010503B was plated on Luria-Bertani (LB) agar by cross-streaking and stored in LB broth with 25% glycerol at -80°C for long-term storage.

### 3.2 Genomic DNA preparation

The genomic DNA (gDNA) was extracted from overnight cultures of the strain 010503B grown in LB broth, utilizing the PureLink^TM^ Genomic DNA Mini Kit in accordance with the manufacturer's instructions. Subsequently, agarose gel electrophoresis and NanoDrop spectrophotometry (Thermo Scientific, USA) were utilized to evaluate the quality of the gDNA.

### 3.3 Whole genome sequencing and assembly

The Nextera XT DNA library preparation kit (Illumina, San Diego, CA, USA) was used to generate sequencing libraries from 1 ng of DNA. The NextSeq 550 sequencer was used to acquire raw sequencing reads using the NextSeq 500/550 high output kit v2.5 (300 cycles, 2 × 150-bp reads) manufactured by Illumina. Quality assessments, adapter trimming, and quality filtering were performed using AfterQC v0.9.6 with the default settings [1]. *De novo* genome assembly was performed using the raw reads and Unicycler v0.5.0 with default settings [2]. The assessment of genome assembly metrics was performed using QUAST v5.0.2, employing the default parameters [3].

### 3.4 Taxonomic identification of the strain

The assessment of genome quality was conducted using CheckM v1.1.2, employing the default settings [4]. The analysis of digital DNA-DNA hybridization (dDDH) and a phylogenomic tree, derived from the whole genome sequences of 010503B and its related type strains, was conducted using the Type (Strain) Genome Server (TYGS) [5].

### 3.5 Genome annotation and antimicrobial gene prediction

A genomic map of 010503B was generated by Proksee [6], and the genome was annotated using the NCBI Prokaryotic Genome Annotation Pipeline (PGAP) with default settings [7]. In addition, whole genome sequence (WGS)-based antimicrobial susceptibility testing was performed using ResFinder v4.3.3 with default settings [8].

## Limitations

The use of next-generation sequencing techniques generates vast quantities of data. However, *de novo* genome assemblies resulting from this data often display significant deficiencies in completeness. The current assemblies possess shortcomings that render them vulnerable to annotation errors, particularly regarding the imprecise estimation of genes that could possibly exist in the draft genome of 010503B.

## Ethics Statement

This work does not involve human or animal subjects and the authors declare that this manuscript is original and has not been published elsewhere.

## CRediT authorship contribution statement

**Uraiwan Kositanont:** Methodology, Data curation, Writing – original draft, Writing – review & editing. **Kanjana Changkaew:** Methodology, Data curation. **Manassanan Phatcharaharikarn:** Methodology, Data curation. **Thunwarat Songngamsuk:** Methodology, Data curation. **Ruchirada Changkwanyeun:** Methodology, Data curation. **Montri Yasawong:** Methodology, Data curation, Writing – original draft, Writing – review & editing, Supervision.

## Data Availability

Draft genome sequence data of the multidrug-resistant bacterium Staphylococcus haemolyticus 010503B isolated from an aerosol sample in a patient waiting area, Thammasat University Hospital, Thailand (Original data) (Genbank).

## References

[1] Chen S., Huang T., Zhou Y., Han Y., Xu M., Gu J. (2017). AfterQC: automatic filtering, trimming, error removing and quality control for fastq data. BMC Bioinform..

[2] Wick R.R., Judd L.M., Gorrie C.L., Holt K.E. (2017). Unicycler: resolving bacterial genome assemblies from short and long sequencing reads. PLoS Comput. Biol..

[3] Mikheenko A., Prjibelski A., Saveliev V., Antipov D., Gurevich A. (2018). Versatile genome assembly evaluation with QUAST-LG. Bioinformatics.

[4] Parks D.H., Imelfort M., Skennerton C.T., Hugenholtz P., Tyson G.W. (2015). CheckM: assessing the quality of microbial genomes recovered from isolates, single cells, and metagenomes. Genome Res..

[5] Meier-Kolthoff J.P., Göker M. (2019). TYGS is an automated high-throughput platform for state-of-the-art genome-based taxonomy. Nat. Commun..

[6] Tatusova T., DiCuccio M., Badretdin A., Chetvernin V., Nawrocki E.P., Zaslavsky L., Lomsadze A., Pruitt K.D., Borodovsky M., Ostell J. (2016). NCBI prokaryotic genome annotation pipeline. Nucleic Acids Res..

[7] Grant J.R., Enns E., Marinier E., Mandal A., Herman E.K., Chen C.Y., Graham M., Van Domselaar G., Stothard P. (2023). Proksee: in-depth characterization and visualization of bacterial genomes. Nucleic Acids Res..

[8] Bortolaia V., Kaas R.S., Ruppe E., Roberts M.C., Schwarz S., Cattoir V., Philippon A., Allesoe R.L., Rebelo A.R., Florensa A.F., Fagelhauer L., Chakraborty T., Neumann B., Werner G., Bender J.K., Stingl K., Nguyen M., Coppens J., Xavier B.B., Malhotra-Kumar S., Westh H., Pinholt M., Anjum M.F., Duggett N.A., Kempf I., Nykäsenoja S., Olkkola S., Wieczorek K., Amaro A., Clemente L., Mossong J., Losch S., Ragimbeau C., Lund O., Aarestrup F.M. (2020). ResFinder 4.0 for predictions of phenotypes from genotypes. J. Antimicrob. Chemother..

